# Reimagining the Biopsychosocial Model: Transdiagnostic Factors to Precision Psychiatry

**DOI:** 10.1016/j.bpsgos.2026.100734

**Published:** 2026-04-17

**Authors:** Maja Nikolic, Charlotte B. Caswell, Nicole Palacio Prada, Maisha Iqbal, Sylvia M.L. Cox, Natalia Jaworska, Natalie Castellanos-Ryan, Frank Vitaro, Mara Brendgen, Sophie Parent, Michel Boivin, Sylvana Côté, Richard E. Tremblay, Jean R. Séguin, Marco Leyton

**Affiliations:** a Integrated Program in Neuroscience, McGill University, Montreal, Quebec, Canada; b Department of Psychology, McGill University, Montreal, Quebec, Canada; c Department of Psychiatry, McGill University, Montreal, Quebec, Canada; d Department of Cellular and Molecular Medicine, University of Ottawa, Ottawa, Ontario, Canada; e University of Ottawa Institute of Mental Health Research, Ottawa, Ontario, Canada; f School of Psychoeducation, Université de Montréal, Montreal, Quebec, Canada; g Azrieli Research Center, Centre Hospitalier Universitaire Sainte-Justine, Montreal, Quebec, Canada; h Department of Psychology, Université de Québec à Montréal, Montreal, Quebec, Canada; i Department of Psychology, Université Laval, Quebec City, Quebec, Canada; j Institute of Genetic, Neurobiological and Social Foundations of Child Development, Tomsk State University, Tomsk, Russia; k Department of Social and Preventative Medicine, Université de Montréal, Montreal, Quebec, Canada; l Departments of Pediatrics and Psychology, Université de Montréal, Montreal, Quebec, Canada; m School of Public Health and Sports Science, University College Dublin, Dublin, Ireland; n Institut National de la Santé et de la Recherche Médicale, U669, Paris, France; o Department of Psychiatry and Addictology, Université de Montréal, Montreal, Quebec, Canada; p Department of Neurology and Neurosurgery, Montreal Neurological Institute, McGill University, Montreal, Quebec, Canada; q Center for Studies in Behavioral Neurobiology, Concordia University, Montreal, Quebec, Canada

**Keywords:** Addiction, Childhood adversity, Diathesis-stress, Dopamine, HiTOP, Neuroimaging

## Abstract

**Background:**

We recently reported evidence that a biopsychosocial model predicts lifetime histories of commonly comorbid early-onset psychiatric disorders. This transdiagnostic model was produced when incorporating either positron emission tomography–measured midbrain dopamine autoreceptors or functional magnetic resonance imaging (fMRI)–measured mesocorticolimbic responses during a monetary incentive delay task. Here, we tested whether a variant of this model incorporating brain responses to alcohol cues specifically predicts future alcohol problems.

**Methods:**

Forty-four youths were assessed longitudinally for externalizing (EXT) behaviors between ages 10 and 16 years, completed fMRI scans and the Childhood Trauma Questionnaire (CTQ) at age 18, and completed the Alcohol Use Disorders Identification Test (AUDIT) at age 25. Binomial logistic regressions were then run with each region of interest.

**Results:**

As hypothesized, the combination of high mesocorticolimbic responses to alcohol cues, high adolescent EXT behaviors, and high CTQ scores predicted higher AUDIT scores 7 years later. Model performance was improved by adding sex as a fourth factor. Significant mesocorticolimbic contributors to the models included the ventral (*p* = .028) and associative striatum (*p* = .017), anterior (*p* = .029) and posterior cingulate (*p* = .005), and ventromedial prefrontal cortex (*p* = .030). All models exhibited good-to-excellent classification performance, with area under the curve values ranging from 0.83 to 0.87, predictive accuracy from 80% to 84%, sensitivity from 74% to 79%, and specificity from 84% to 92%.

**Conclusions:**

Together, this work delineates a novel hierarchical model with precision psychiatry factors superimposed on transdiagnostic liability. High mesocorticolimbic reactivity to specific stimuli might shape the expression of psychopathology.

## Plain Language Summary

In a longitudinally followed cohort, we tested whether future alcohol use problems can be predicted by the combination of transdiagnostic risk factors and brain responses to alcohol-specific cues. As hypothesized, higher adolescent externalizing propensities, higher childhood trauma, and higher mesocorticolimbic responses predicted more alcohol problems 7 years later. The results support our recently proposed hierarchical model where mental health problems arise from disorder-specific factors superimposed upon a general liability.

Accumulating epidemiological evidence suggests that many mental health disorders arise from issue-specific susceptibilities superimposed upon a general liability to diverse problems (1,2). The processes shaping this dynamic remain little explored, but a few factors have been implicated with some consistency.

One of the best-established transdiagnostic factors is early-life adversity (3, 4, 5). Prospective and quasi-experimental studies suggest that early trauma not only covaries with nearly all forms of psychopathology but also makes a causal contribution (6). Despite this, only some individuals exposed to even severe adversity develop mental health disorders (7).

Diverse disorders are also predicted by dysregulated adolescent emotions, behavioral control, and mesocorticolimbic reactivity (8, 9, 10, 11, 12). However, these effects have also not been large when studied in isolation. One possibility is that these factors interact in a diathesis-stress process, whereby preexisting vulnerabilities (e.g., externalizing [EXT] behaviors or brain reactivity) moderate the psychological consequences of early-life adversity. Indeed, there is evidence that the negative consequences of stressful events are increased in individuals exhibiting dysregulated mesocorticolimbic reactivity and adolescent EXT behaviors (i.e., impulsivity and outwardly directed emotional turbulence) (12, 13, 14, 15, 16).

Recently, we tested whether combining all 3 of the above factors would yield a particularly powerful biopsychosocial model. In our first test of this hypothesis, a combination of high early-life trauma, high adolescent EXT behaviors, and low midbrain dopamine D_2_ receptor availability (putative autoreceptors) identified participants with lifetime histories of commonly comorbid early-onset disorders with strikingly high statistical robustness, sensitivity, and specificity (17). Since then, we have reproduced this model in a larger sample (*N* = 1338 vs. 52) using functional magnetic resonance imaging (fMRI) data instead of positron emission tomography (PET) (18). Reward-related neural signals were derived from mesocorticolimbic regions of interest (ROIs), and reward anticipation was indexed using the monetary incentive delay (MID) task. As predicted, the 3-factor biopsychosocial model was statistically powerful with high levels of sensitivity and accuracy; within this model, reward anticipation responses in the ventral striatum (VS), caudate, putamen, and anterior cingulate cortex (ACC) at age 14 identified who had a mental health disorder by age 19. Moreover, the effect of childhood trauma was moderated by responses in the striatum and ACC such that low responses at age 14 strengthened the association between early-life adversity and psychopathology by age 19, whereas high responses appeared to buffer against this risk. Together, these findings tentatively identify a novel diathesis-stress model, wherein the vulnerable diathesis (brain reactivity to reward-related cues) affects responses to early-life trauma.

The shaping of this hypothesized general liability into a specific problem might reflect the development of large brain reward responses to disease-specific stimuli. For example, people with substance use disorders (SUDs) exhibit blunted mesocorticolimbic responses during monetary reward anticipation tasks but high responses to addiction-related cues (19,20). Unclear, however, is whether the latter responses are a preexisting vulnerability trait or a consequence of extensive substance use. Compared with healthy control participants, people at high risk for SUDs have been reported to show high (21,22), low (22,23), and undifferentiated brain responses to addiction-related cues (24). Since a clearer picture might emerge after controlling for the effects of early-life trauma and EXT behaviors, we tested here whether a variant of our biopsychosocial model incorporating mesocorticolimbic responses to alcohol cues would identify youths who develop higher alcohol use problems at a 7-year follow-up.

## Methods and Materials

### Participants

Participants (*N* = 57; mean ± SD age = 18.5 ± 0.6 years) who drank alcohol and had been followed since birth were recruited from the QLSCD (Quebec Longitudinal Study of Child Development) (*n* = 51) and the QNTS (Quebec Newborn Twin Study) (*n* = 6). At approximately age 18 years, all completed fMRI (25), and 47 underwent [^18^F]fallypride (17,26) and [^11^C]ABP688 PET scans (27). For the current analyses with fMRI data, 13 participants were excluded due to missing follow-up data 7 years later, leaving a final sample of 44 (26 female/18 male). Mean age at follow-up assessing alcohol use was 25.7 ± 0.6 years. Participants classified as high Alcohol Use Disorders Identification Test (AUDIT) were defined as those scoring equal to or above the sample’s median score at follow-up (≥5). Ethics approval was obtained from McGill University and Sainte-Justine University Hospital Research Ethics Boards, and all participants provided written informed consent.

### EXT Scores

Adolescent EXT behaviors (e.g., impulsivity, aggression) were measured annually from ages 10 to 16 using the Social Behavior Questionnaire (28,29). Based on their average EXT score during this period, individuals from the first wave of the QLSCD cohort (*n* = 242) were selected from the upper and lower 30% (30).

### Screening and Assessments

At approximately 18 years of age, participants completed the Structured Clinical Interview for DSM-5 (31), the Timeline Follow-Back Method to assess drug and alcohol use (32), and the AUDIT (33). Quantifications of alcohol use across years were obtained for the total number of use occasions (lifetime alcohol use), intoxication occasions (defined as subjectively feeling intoxicated after consuming ≥2 drinks), and binge occasions (≥4 drinks for females, ≥5 drinks for males per occasion). The Childhood Trauma Questionnaire (CTQ) (34) assessed childhood abuse and neglect across 5 domains: emotional abuse, physical abuse, sexual abuse, emotional neglect, and physical neglect. All clinical assessments were repeated 7 years later at approximately 25 years of age. Participants (*N* = 44) were classified as having higher versus lower alcohol use problems based on a median split of follow-up AUDIT scores (≥5).

### Alcohol Visual-Taste Cue Paradigm

As described in more detail elsewhere (25), participants at baseline (i.e., ∼18 years) were presented with visual-gustatory cues in an fMRI task involving 1) an alcohol tastant (0.5 mL of their preferred alcoholic beverage mixed with 0.5 mL of juice), 2) juice (1 mL of their favorite juice as a nondrug appetitive control), and 3) water (1 mL as a low-motivational control). All participants selected orange juice alone and vodka with orange juice as their preferred beverage cues, respectively. Each trial consisted of a 1-second image, 1-second spray delivery, 2-second swallow prompt, and 12-second image presentation. Rinses and fixation trials followed each cue. Cues were pseudorandomized across 39 trials (i.e., 13 per tastant). This corresponded to approximately 0.158 grams ethanol per alcohol delivery (approximately 0.0024 g/kg at the sample’s mean body weight).

### MRI Acquisition

#### Magnetic Resonance Imaging

Participants underwent 1 MRI session at baseline, including anatomical and fMRI acquisitions. Scans were acquired on a 3T Siemens Trio MRI scanner (12-channel head coil). T1-weighted images were acquired using a magnetization-prepared rapid gradient-echo (MPRAGE) sequence (TI/TR/TE = 900/2300/4.18 ms; 1-mm^3^ voxels). Echo*-*planar imaging fMRI was angled 30° off the anterior commissure*–*posterior commissure plane to reduce dropout (TR = 2330 ms, voxel size = 3 mm^3^, 42 slices).

#### fMRI Analysis

fMRI data were preprocessed using SPM12 (Wellcome Department of Cognitive Neurology) on MATLAB R2022a (version 9.12; The MathWorks, Inc.). Preprocessing included manual reorientation to the Montreal Neurological Institute (MNI) space, slice-timing correction, realignment, coregistration to anatomical images, segmentation, normalization, and 8-mm full width at half maximum smoothing. Motion thresholds were set at <4-mm translation and <4° rotation (those exceeding these were excluded).

Visual-gustatory cues were modeled in 16-second blocks, including the full delivery of the gustatory stimulus and accompanying image presentation. First-level generalized linear models were used to model condition-specific contrasts. Six contrasts were generated per participant: alcohol > juice, alcohol > water, juice > water, water > fixation, juice > fixation, and alcohol > fixation. For the current analyses, the focus was on the alcohol > juice and alcohol > water contrasts.

### ROI Analysis

As described previously (25), a priori bilateral ROIs included the VS, associative striatum (AS), sensorimotor striatum (SS), amygdala, ACC, dorsolateral prefrontal cortex (dlPFC), ventromedial PFC (vmPFC), posterior cingulate cortex (PCC), insula, and substantia nigra (SN)/ventral tegmental area (VTA). All ROIs were defined independently of the primary analysis (Supplement). Beta values for each ROI and contrast were extracted using SPM12 and entered into IBM SPSS Statistics (version 29.0.1.1; IBM Corp.) for further analyses. In secondary analyses, a composite ROI was created by averaging the 10 ROI beta values (then *z* scoring), and this single measure was used as the neural predictor.

### Statistical Analyses

#### Outcomes (Y)

The primary outcome was follow-up alcohol use group at age 25 (AUDIT ≤4 vs. ≥5). The secondary outcome was continuous follow-up AUDIT scores at age 25. Additional models examined the presence of DSM-5 diagnoses at follow-up.

#### Predictors (X)

Predictors included ROI-specific alcohol cue–evoked blood oxygen level–dependent (BOLD) responses at age 18 (alcohol vs. juice; with alcohol vs. water examined for generalizability), CTQ scores at age 18, and EXT behavior scores (ages 10–16); all predictors were *z* scored. Sex, baseline AUDIT score at age 18, and a composite ROI variable were examined in additional models.

#### Binary Logistic Regression

Binary logistic regressions tested whether a 3-factor model—comprising alcohol versus juice BOLD responses at age 18, CTQ scores at age 18, and EXT scores at ages 10 to 16 (all *z* scored)—predicted higher versus lower AUDIT group membership at follow-up (i.e., 7 years after the initial fMRI and clinical assessments). Ten separate logistic regressions were run, each using a different ROI as the neural predictor. The same modeling approach was applied using the alcohol versus water contrast to assess generalizability across control conditions. Sex was tested as a fourth factor in follow-up models to determine whether its inclusion further improved model fit. In additional models, AUDIT scores at age 18 were included as a fourth factor to evaluate whether mesocorticolimbic BOLD responses to alcohol cues predicted future alcohol use above and beyond prior alcohol consumption at that developmental stage. Additional analyses tested a 3-factor model using a composite ROI score as the neural predictor, along with CTQ scores at age 18 and EXT behavior scores (ages 10–16). Parallel analyses tested whether the alcohol versus juice 3-factor models predicted the presence of DSM-5 diagnoses at follow-up.

Given the sample size, coefficients and 95% CIs were also estimated using nonparametric bootstrapping (1000 resamples; percentile method) (35). Linearity of the logit was assessed with the Box-Tidwell test; no violations were observed (all *p*s ≥ .232) (36). Receiver operating characteristic (ROC) curves were generated for each model. Sensitivity was plotted against 1 − specificity, and area under the curve (AUC) summarized overall discrimination (higher values indicate better discrimination; 0.5 indicates chance) (37). Nested models (1- to 4-factor) were compared using likelihood ratio tests to evaluate incremental fit from CTQ scores, ROI BOLD, and sex, depending on the outcome (37). Additional methodological details are provided in the Supplement.

#### Linear Regressions

Multiple linear regressions tested whether the same 3-factor model (ROI-specific alcohol vs. juice BOLD responses at age 18, CTQ scores at age 18, and EXT behavior scores at ages 10–16; all *z* scored) predicted continuous AUDIT scores at follow-up (7 years later). Ten separate regressions were run, each using a different ROI as the neural predictor. Sex was added in follow-up models to evaluate whether this variable explained additional variance beyond the 3-factor model. Additional models tested the same model using the composite ROI.

For continuous follow-up AUDIT, nested linear regressions were compared using Δ*R*^2^ and *F* change. For each ROI, a 2-factor model (CTQ+EXT) was compared with a 3-factor model (CTQ+EXT+ROI BOLD), and sex was added in follow-up models; improvement was assessed relative to the corresponding reduced model (*p* < .05) (38).

#### Group Differences in Alcohol-Cue Reactivity: ROI Analysis of Covariance Controlling for EXT and CTQ Scores

Finally, a 2-way group × ROI analysis of covariance (ANCOVA) with EXT behavior and CTQ scores as covariates was used to compare alcohol versus juice BOLD responses in participants with higher versus lower AUDIT scores 7 years later.

All analyses were performed using IBM SPSS Statistics version 29.0.1.1.

## Results

Participants were 18.4 ± 0.54 and 25.7 ± 0.6 years of age at the time of the fMRI scan and 7-year follow-up interview, respectively. The 2 subgroups (high vs. low AUDIT scores at age 25) had similar alcohol use indices at age 18, but by design, they differed at age 25 (Table 1).

**Table 1:** Demographic Characteristics of Study Participants (*N=* 44)

	Total Sample	Low AUDIT, *n* = 25	High AUDIT, *n* = 19	*p* Value
Female/Male	26/18	18/7	8/11	χ^2^_1_ = 3.991, *p* = .046
Age at fMRI Scan, Years	18.39 ± 0.54	18.36 ± 0.57	18.42 ± 0.51	.714
Age at 7-Year Follow-Up	25.57 ± 0.62	25.56 ± 0.58	25.58 ± 0.69	.922
EXT Scores	1.3 ± 0.97	1.00 ± 0.89	1.68 ± 0.97	.021∗
CTQ				
Total	32.23 ± 5.89	31.08 ± 3.62	33.74 ± 7.83	.14
Emotional abuse	6.2 ± 2.6	5.72 ± 1.21	6.84 ± 3.70	.22
Physical abuse	5.23 ± 0.64	5.20 ± 0.71	5.26 ± 0.56	.75
Sexual abuse	5.09 ± 0.6	5.00 ± 0	5.21 ± 0.92	.33
Emotional neglect	8.91 ± 3.46	8.60 ± 3.23	9.32 ± 3.74	.50
Physical neglect	5.80 ± 1.36	5.40 ± 1.04	6.32 ± 1.57	.035∗
Minimization/denial	1.00 ± 1.18	1.16 ± 1.25	0.79 ± 1.08	.31
Baseline				
AUDIT scores	5.30 ± 3.59	4.40 ± 3.24	6.47 ± 3.77	.057
Number of alcohol uses	98.3 ± 120.8	73.4 ± 91.9	131.2 ± 146.9	.117
Intoxication occasions	47.2 ± 48.6	31.4 ± 31.1	67.9 ± 59.5	.022∗
Binge occasions	32.8 ± 42.5	21.3 ± 27.4	48.0 ± 53.7	.059
7-Year Follow-Up				
AUDIT scores	5.0 ± 3.5, median = 4.0, skewness = 1.020	2.6 ± 1.4	8.2 ± 2.9	<.001∗
Number of lifetime alcohol uses	649.2 ± 532.3	358.5 ± 261.0	1016.3 ± 564.9	<.001∗
Number of intoxication occasions	267.0 ± 303.9	149.3 ± 136.6	415.7 ± 387.0	.005∗
Number of binge occasions	129.1 ± 149.5	83.3 ± 88.5	187.0 ± 189.2	.037∗
Cannabis occasions	345.8 ± 1122.8	304.2 ± 1308.6	403.5 ± 831.4	.78
Non-THC drug use occasions	52.9 ± 257.3	73.8 ± 337.1	25.5 ± 72.1	.54
Lifetime DSM-5 Diagnoses				
No disorder	19 (43.2%)	10 (40%)	9 (47.4%)	.856
EXT disorder	4 (9.1%)	3 (12%)	1 (5.3%)	
INT disorder	13 (29.5%)	7 (28%)	6 (31.6%)	
EXT+INT disorders	8 (18.2%)	5 (20%)	3 (15.8%)	

Values are reported as mean ± SD, *n*, or *n* (%) unless otherwise reported.

∗*p* < .05.

AUDIT, Alcohol Use Disorders Identification Test; CTQ, Childhood Trauma Questionnaire; EXT, externalizing; fMRI, functional magnetic resonance imaging; INT, internalizing.

### Primary Outcome (Dichotomous Follow-Up AUDIT)

#### Three-Factor ROI Models (EXT+CTQ+Alcohol vs. Juice BOLD) Predicting Higher Versus Lower AUDIT Group

In the logistic regression models, higher versus lower AUDIT group membership was predicted by the combination of CTQ scores, EXT behavior scores, and age 18 alcohol versus juice cue-evoked BOLD responses in 9 of the 10 ROIs (Table 2 and Table S1). Significant ROI effects were observed in the AS (*p* = .036), VS (*p* = .051), vmPFC (*p* = .039), and PCC (*p* = .007) (Table 2 and Table S1). The strongest model incorporated PCC responses, correctly classifying 75% of the cases and accounting for 42.2% of the variance in AUDIT group membership (Nagelkerke *R*^2^). Model performance was high (sensitivity = 73.7%, specificity = 76%, positive predictive value = 70%, negative predictive value = 79.2%, ROC AUC = 0.821). In this model, each 1-SD increase in PCC response was associated with a 5-fold higher odds of higher follow-up AUDIT group membership. Performance across other ROIs was similar (Table 2 and Table S1).

**Table 2:** Three-Factor Binomial Regression Models With Each Region of Interest for the Alcohol Versus Juice Cue Contrast Predicting Future High Alcohol Use

	Alcohol vs. Juice BOLD		EXT		CTQ		Full Model	AUC (95% CI)	Classification Predictive Accuracy, Sensitivity, and Specificity
	*p*	OR (95% CI)	*p*	OR (95% CI)	*p*	OR (95% CI)	χ^2^, *p*, Nagelkerke *R*^2^		
PCC	.007	5.55 (1.613–19.112)	.018	3.09 (1.21–7.92)	.42	1.43 (0.59–3.43)	16.6, *p* < .001, 0.42	0.821 (0.698–0.944)	75%, 73.7%, and 76%
AS	.036	3.377 (1.085–10.507)	.026	2.62 (1.13–6.08)	.35	1.57 (0.61–4.02)	12.84, *p* = .005, 0.34	0.798 (0.669–0.926)	63.6%, 52.6%, and 72%
vmPFC	.039	2.756 (1.053–7.213)	.031	2.55 (1.09–5.98)	.27	1.64 (0.68–3.97)	12.09, *p* = .007, 0.32	0.785 (0.648–0.922)	70.5%, 57.9%, and 80%
VS	.051	3.267 (0.996–10.718)	.035	2.47 (1.07–5.71)	.287	1.65 (0.66–4.13)	11.82, *p* = .008, 0.32	0.789 (0.658–0.921)	63.6%, 47.4%, and 76%
SS	.157	1.861 (0.787–4.398)	.039	2.355 (1.043–5.315)	.330	1.539 (0.647–3.662)	8.636, *p* = .035, 0.239	0.733 (0.582–0.883)	68.2%, 52.6%, and 80%
Amygdala	.205	1.747 (0.737–4.143)	.043	2.223 (1.024–4.826)	.331	1.566 (0.634–3.871)	8.154, *p* = .043, 0.227	0.728 (0.575–0.882)	65.9%, 42.1% and 84%
SN/VTA	.262	1.541 (0.723–3.281)	.045	2.193 (1.019–4.717)	.373	1.520 (0.605–3.820)	7.720, *p* = .052, 0.216	0.716 (0.556–0.875)	65.9%, 47.4%, and 80%
dlPFC	.074	2.482 (0.916–6.726)	.038	2.277 (1.047–4.954)	.369	1.514 (0.612–3.745)	11.031, *p* = .012, 0.298	0.777 (0.640–0.913)	68.2%, 57.9%, and 76%
ACC	.069	2.635 (0.927–7.488)	.027	2.589 (1.117–6.004)	.340	1.558 (0.626–3.875)	11.021, *p* = .012, 0.297	0.768 (0.631–0.906)	63.6%, 52.6%, and 72%
Insula	.422	1.398 (0.617–3.168)	.045	2.201 (1.018–4.760)	.301	1.579 (0.664–3.752)	7.083, *p* = .069, 0.200	0.712 (0.554–0.869)	70.5%, 57.9%, and 80%

ACC, anterior cingulate cortex; AS, associative striatum; AUC, area under the curve; BOLD, blood oxygen level–dependent; CTQ, Childhood Trauma Questionnaire; dlPFC, dorsolateral prefrontal cortex; EXT, externalizing; OR, odds ratio; PCC, posterior cingulate cortex; SN, substantia nigra; SS, sensorimotor striatum; vmPFC, ventromedial prefrontal cortex; VS, ventral striatum; VTA, ventral tegmental area.

#### Composite ROI Model

A 3-factor model using the composite ROI score also distinguished higher versus lower follow-up AUDIT group membership (model χ^2^_3_ = 12.166, *p* = .007). In the model, the composite ROI term (*p* = .043, odds ratio [OR] = 3.946) and EXT behavior scores (*p* = .026, OR = 2.670) were significant, whereas CTQ scores were not (*p* = .313, OR = 1.617) (Table S2).

#### Incremental Contribution of CTQ (Model Fit)

Although CTQ was not a significant independent predictor in these 3-factor models, likelihood ratio (LR) tests indicated that adding CTQ scores significantly improved model fit beyond EXT scores and ROI response for models incorporating responses from the AS (*p* = .025), VS (*p* = .025), SS (*p* = .045), amygdala (*p* = .032), SN/VTA (*p* = .033), dlPFC (*p* = .031), insula (*p* = .046), ACC (*p* = .039), and vmPFC (*p* = .045) (Table S3). Similarly, in the composite ROI model, adding CTQ scores to the composite ROI and EXT behavior model significantly improved model fit (Δ−2LL = 4.266, LR χ^2^_1_ = 4.266, *p* = .039) (Table S4).

### Sensitivity Analysis: 4-Factor Models Including Baseline AUDIT Scores

Given the potential importance of baseline alcohol use, we tested the effect of including AUDIT scores at age 18 as a fourth factor (EXT behavior, CTQ, ROI response, and baseline AUDIT). Baseline AUDIT scores were not a significant independent predictor of lower versus higher alcohol use at follow-up in any ROI model (all *p*s ≥ .373). Key ROI effects remained significant in the PCC (*p* = .009), AS (*p* = .048), and vmPFC (*p* = .048), whereas the composite ROI effect was trend level (*p* = .065) (Tables S5 and S6).

#### Four-Factor Models Including Sex (EXT+CTQ+ROI Response+Sex)

Because sex differences in alcohol use are well established, sex was added as a fourth factor (39,40). Adding sex significantly improved model fit relative to the 3-factor models (Table 3 and Table S7). After controlling for EXT behavior, CTQ, and sex, ROI response effects remained significant in the PCC (*p* = .005), AS (*p* = .017), VS (*p* = .028), vmPFC (*p* = .030), and ACC (*p* = .029) (Table 3). In these 4-factor models, sex was a significant independent predictor (*p* < .046, OR range = 0.12–0.20), indicating higher odds of higher alcohol use in men (Table 3). The 4-factor models correctly classified up to 84.1% of cases and explained more than 42% of the variation in alcohol use. The 4-factor models demonstrated higher sensitivity compared with the 3-factor models (Table 3).

**Table 3:** Binomial Regression Models for the Alcohol Versus Juice Contrast, Including Sex as a Predictor of Future High Alcohol Use

	Alcohol vs. Juice BOLD		EXT		CTQ		Sex		Full Model	AUC (95% CI)	Classification Predictive Accuracy, Sensitivity, and Specificity
	*p*	OR (95% CI)	*p*	OR (95% CI)	*p*	OR (95% CI)	*p*	OR (95% CI)	χ^2^, *p*, Nagelkerke *R*^2^		
PCC	.005	6.233 (1.75–22.13)	.019	3.11 (1.20–8.05)	.47	1.40 (0.57–3.48)	.046	0.19 (0.038–0.97)	20.99, *p* < .001, 0.51	0.867 (0.757–0.977)	79.5%, 73.7%, and 84%
AS	.017	6.63 (1.40–31.29)	.029	2.74 (1.11–6.76)	.36	1.52 (0.62–3.70)	.022	0.123 (0.020–0.74)	19.21, *p* < .001, 0.48	0.865 (0.755–0.976)	79.5%, 73.7%, and 84%
vmPFC	.030	3.41 (1.126–10.336)	.034	2.56 (1.08–6.1)	.32	1.56 (0.64–3.81)	.042	0.201 (0.043–0.95)	16.64, *p* = .002, 0.42	0.832 (0.700–0.963)	84.1%, 73.7%, and 92%
VS	.028	5.91 (1.209–28.88)	.040	2.55 (1.04–6.21)	.272	1.68 (0.66–4.22)	.027	0.15 (0.028–0.804)	17.55, *p* = .002, 0.44	0.863 (0.749–0.977)	81.8%, 78.9%, and 84%
ACC	.029	5.0 (1.18–21.19)	.024	2.84 (1.15–7.03)	.39	1.46 (0.61–3.48)	.024	0.134 (0.023–0.77)	17.14, *p* = .002, 0.43	0.842 (0.717–0.967)	79.5%, 73.7%, and 84%
SS	.068	2.42 (0.94–6.23)	.036	2.52 (1.06–5.99)	.38	1.47 (0.62–3.47)	.033	0.192 (0.042–0.87)	13.673, *p* = .008, 0.358	0.827 (0.700–0.955)	77.3%, 68.4%, and 84%
Amygdala	.104	2.18 (0.852–5.606)	.041	2.37 (1.04–5.41)	.403	1.44 (0.61–3.43)	.038	0.209 (0.048–0.919)	12.824, *p* = .012, 0.339	0.802 (0.664–0.940)	77.3%, 73.7%, and 80%
SN/VTA	.076	2.191 (0.920–5.213)	.038	2.494 (1.05–5.91)	.525	1.33 (0.55–3.22)	.028	0.169 (0.035–0.822)	13.202, *p* = .010, 0.348	0.821 (0.683–0.959)	81.8%, 73.7%, and 88%
dlPFC	.080	2.582 (0.892–7.471)	.042	2.316 (1.03–5.19)	.459	1.382 (0.587–3.251)	.086	0.282 (0.067–1.196)	14.114, *p* = .007, 0.368	0.832 (0.704–0.959)	81.8%, 73.7%, and 88%
Insula	.299	1.597 (0.661–3.862)	.040	2.323 (1.04–5.19)	.374	1.46 (0.634–3.357)	.056	0.254 (0.062–1.04)	10.941, *p* = .027, 0.295	0.802 (0.663–0.941)	77.3%, 73.7%, and 80%

ACC, anterior cingulate cortex; AS, associative striatum; AUC, area under the curve; BOLD, blood oxygen level–dependent; CTQ, Childhood Trauma Questionnaire; dlPFC, dorsolateral prefrontal cortex; EXT, externalizing; OR, odds ratio; PCC, posterior cingulate cortex; SN, substantia nigra; SS, sensorimotor striatum; vmPFC, ventromedial prefrontal cortex; VS, ventral striatum; VTA, ventral tegmental area.

#### The Models Are Reproduced Using Bootstrapping

The primary 3- and 4-factor findings were unchanged with bootstrapping (Tables S23 and S24).

### Secondary Outcome: Continuous Follow-Up AUDIT Scores

As exploratory analyses, parallel linear regression change models tested whether alcohol versus juice BOLD response explained incremental variance in continuous follow-up AUDIT scores beyond the effects of EXT behavior and CTQ scores. Across ROIs, the 3-factor models were significant overall (all *p*s ≤ .013) and explained 23% to 33% of the variance in follow-up AUDIT scores (Table S8). CTQ scores emerged as a significant independent predictor across all ROI models (*p* ≤ .018), whereas EXT scores reached statistical significance in the models incorporating PCC (*p* = .036), AS (*p* = .049), and ACC (*p* = .050) responses and trend-level significance in the remaining ROIs (Table S8).

Only PCC response explained incremental variance beyond EXT behavior and CTQ scores (Δ*R*^2^ = 0.103, *p* = .017) and predicted follow-up AUDIT scores in the full model (β = 0.323, *p* = .017) (Tables S8 and S9). Adding sex did not improve model fit (Δ*R*^2^ = 0.010, *p* = .444) (Table S10).

A parallel 3-factor model using the composite mesocorticolimbic ROI score was significant overall (*p* = .005, *R*^2^ = 0.275) (Table S11). EXT behavior (β = 0.286, *p* = .048) and CTQ (β = 0.358, *p* = .014) scores were significant predictors, whereas the composite ROI score was not (β = 0.218, *p* = .119) and did not improve fit beyond EXT+CTQ (Δ*R*^2^ = 0.047, *p* = .115) (Tables S11 and S12).

### Additional Analyses

#### Binomial Regressions: Presence or Absence of DSM-5 Disorders at Follow-Up

EXT behavior scores alone predicted DSM-5 disorders 7 years later (*p* = .050, OR = 2.059) (Table S13). Adding ROI alcohol versus juice responses and CTQ scores did not improve model fit (all *p*s ≥ .145) (Table S16).

#### Higher Alcohol-Cue BOLD Responses in Participants With High AUDIT 7 Years Later

**Figure 1:**
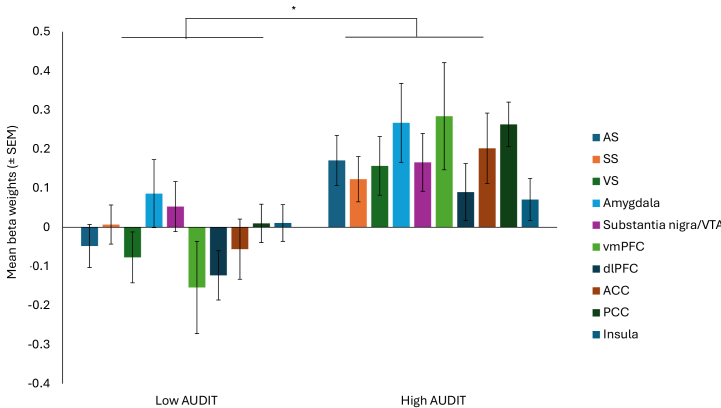
Mean beta weights for the alcohol vs. juice contrast in all 10 regions of interest, controlling for externalizing and Childhood Trauma Questionnaire scores. The high Alcohol Use Disorders Identification Test (AUDIT) group showed greater mesocorticolimbic responses in the alcohol vs. juice contrast compared with the low AUDIT group (*p* = .020). ∗*p* < .05, high vs. low AUDIT groups. ACC, anterior cingulate cortex; AS, associative striatum; dlPFC, dorsolateral prefrontal cortex; PCC, posterior cingulate cortex; SS, sensorimotor striatum; vmPFC, ventromedial prefrontal cortex; VS, ventral striatum; VTA, ventral tegmental area.

Alcohol versus juice contrast BOLD responses at age 18 were not significantly different in participants with higher versus lower AUDIT scores in the analysis of variance without EXT behavior and CTQ scores included as covariates (*F*_1,42_ = 3.19, *p* = .081), but this effect did emerge when EXT behavior and CTQ scores were controlled for (*F*_1,40_ = 5.91, *p* = .020). The absence of a significant group × ROI interaction (*F*_3.69,147.57_ = 1.89, *p* = .121) indicated that the effect was most conservatively interpreted as occurring across all 10 ROIs (Figure 1). Individual differences in alcohol versus juice BOLD responses were not accounted for by indices of alcohol use at age 18 (i.e., number of alcohol use occasions, intoxications, and binges) (*r* ≤ 0.083, *p* ≥ .201).

## Discussion

**Figure 2:**
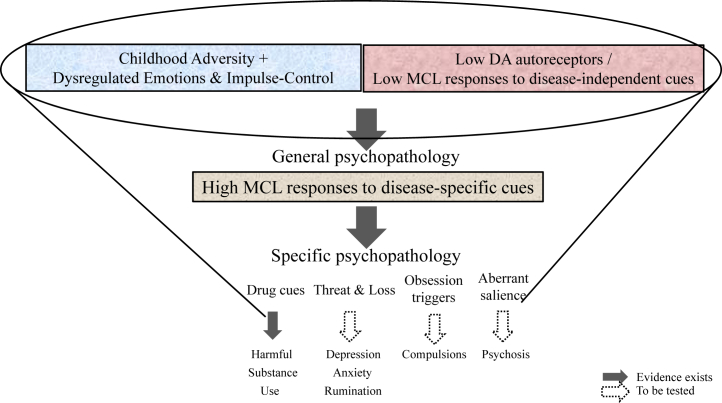
Hierarchical diathesis-stress model. The model’s structure is influenced by the Hierarchical Taxonomy of Psychopathology (1), with proposed mechanisms based on our 3 recent studies [(17,18) and the current study]. Together, they suggest that a broad liability to diverse psychopathology is promoted by a combination of 1) early-life trauma, 2) dysregulated emotional and impulse control, and 3) poorly regulated mesocorticolimbic (MCL) reactivity as indexed by low dopamine (DA) autoreceptor availability and low MCL responses to disease-independent cues. Specific disorders are predicted by high MCL responses to disease-specific cues after controlling for the first 2 factors. The solid arrows identify pathways with empirical support.

This is the third in a series of studies demonstrating that the combination of early-life adversity, adolescent EXT behaviors, and dysregulated mesocorticolimbic function identifies clinical outcomes with high statistical robustness and accuracy. In our previous studies, the incorporated biological variables were PET-measured dopamine autoreceptors (17) and fMRI-measured reward anticipation responses during a MID task (18). Both models had transdiagnostic power, identifying all commonly comorbid early-onset psychiatric disorders. Here, we report that a variant of the model incorporating high mesocorticolimbic responses to alcohol cues at age 18 specifically predicted the likelihood of higher alcohol use at age 25. Together, these studies tentatively identify factors that shape a biopsychosocial model with both transdiagnostic and precision psychiatry levels (Figure 2).

Our previous fMRI study suggests that the model is consistent with a diathesis-stress perspective (18). The lower the brain responses to disease-independent reward cues, the greater the effect of early-life adversity on clinical outcomes (18). The current study reimagines this model as a hierarchy (1) wherein elevated mesocorticolimbic reactivity to disease-related cues (e.g., alcohol cues) shapes susceptibility to a specific problem (e.g., harmful alcohol use). Similar effects are hypothesized for mood and anxiety disorders when incorporating heightened responses to aversive stimuli (Figure 2).

Consistent with the multifactorial regression analyses, participants who had higher AUDIT scores at age 25 showed larger alcohol cue–induced BOLD responses at age 18 across mesocorticolimbic ROIs. Critically, this effect was evident only after accounting for early-life adversity and adolescent EXT behavior. Potentially reflecting the importance of controlling for these additional influences, high alcohol cue–induced brain responses have not been observed consistently in studies of high-risk youths (21, 22, 23, 24, 25,41,42). If alcohol cues come to potently activate mesocorticolimbic pathways over and above the effects of EXT behavior and adversity, the high-risk state might then transition to a frank alcohol use disorder (19,43). At this time, the high drug cue–induced brain response may serve as a biomarker (44).

Within the current study’s multifactorial regression models, future higher alcohol use was predicted by greater alcohol versus juice cue responses in the VS, AS, ACC, PCC, and vmPFC. Each of these regions has been linked to reward-seeking behaviors. The VS and AS influence reward processing, conditioned approach, and habit formation (45, 46, 47, 48). The vmPFC is implicated in executive function, including valuation, self-control, and salience attribution (49,50). The cingulate integrates cognitive, motor, and motivational processes (51). In addition, a composite ROI score capturing mesocorticolimbic cue reactivity across regions supported the same pattern at the network level. In the 3-factor model, the composite ROI predictor significantly differentiated higher versus lower follow-up AUDIT outcomes, consistent with the view that broader mesocorticolimbic activation, rather than any single region, is informative of risk (20).

Several regions involved in reward and salience processing, including the SS, SN/VTA, and insula, did not emerge as significant individual predictors of high alcohol use in the binomial regression analyses. This might reflect a lack of statistical power. Indeed, the ANCOVA failed to identify a group × ROI interaction. This raises the possibility that the most conservative interpretation is that all tested ROIs contributed equally. This noted, engagement of the dorsolateral striatum typically becomes more prominent as substance use transitions from goal-directed to compulsive behaviors, whereas the VS and AS are more commonly implicated in early-stage, motivational processes (47,52). At age 18, when the fMRI data were collected, the current sample was at risk for, but not yet engaged in, heavy alcohol use.

Insula responses at age 18 in the binomial regressions also failed to differentiate groups. This was unexpected as even a small-volume alcohol tastant cue is likely to engage interoceptive awareness (43). One possibility is that insula’s contribution to problematic alcohol use is more context specific, emerging under conditions of heightened craving, risk, or motivational conflict (53), which were not explicitly elicited in our task. Finally, only trend-level effects were seen in the SN/VTA ROIs (Table 3), plausibly reflecting limitations in BOLD signal sensitivity in deep brain structures (54).

In these models, baseline AUDIT score was not an independent predictor of follow-up alcohol use group (high vs. low; all *p*s ≥ .373). However, alcohol-cue reactivity in the PCC, AS, and vmPFC predicted high alcohol use at follow-up after adjusting for baseline AUDIT scores, suggesting that cue reactivity explains variance in later alcohol use beyond the effects of baseline alcohol use severity.

In secondary analyses using continuous follow-up AUDIT scores, the 3-factor model (EXT behavior, CTQ, and alcohol cue–induced BOLD response) showed reduced regional specificity, with only the PCC BOLD term predicting follow-up AUDIT scores beyond the effects of EXT behavior and CTQ scores. Notably, CTQ scores predicted follow-up AUDIT scores across all ROI models, consistent with evidence linking childhood trauma to adolescent alcohol use and misuse (55,56).

Adolescent EXT behaviors alone predicted DSM-5 disorders 7 years later, whereas adding alcohol cue–evoked BOLD responses did not improve model fit. These findings are consistent with epidemiological research suggesting that EXT behaviors reflect a broad developmental liability to diverse mental health problems (10,30,57, 58, 59, 60, 61). These adolescent EXT behavior propensities may be aggravated by childhood adversity, suggesting a pathway by which environmental stress amplifies risk for mental health disorders (18,62,63). In comparison, the inability of alcohol cue–induced BOLD responses to improve the prediction of diverse mental health problems strengthens the proposition that this response specifically predicts future alcohol use.

As expected, males were more likely to exhibit higher alcohol use. Including sex as a fourth factor strengthened model fit and enhanced the predictive contribution of each ROI. These 4-factor models demonstrated excellent sensitivity, correctly classifying up to 80% of the cases and explaining more than 42% of the variance in alcohol use problems. These findings highlight the importance of considering sex differences when identifying neurobiological and behavioral risk factors. They are also consistent with epidemiological evidence that males are more likely than females to engage in heavy drinking and meet criteria for an alcohol use disorder (35,40).

### Strengths and Limitations

A key strength of this study is the use of fMRI in a longitudinally followed birth cohort, allowing for a prospective investigation of clinical outcomes. To date, elevated mesocorticolimbic alcohol-cue reactivity has been linked to subsequent drinking escalation in only one prior study, and the follow-up interval was limited to 1 year (21). Here, higher mesocorticolimbic responses to alcohol cues at baseline predicted higher alcohol use 7 years later after accounting for early-life adversity and adolescent EXT behaviors. This pattern supports the potential relevance of alcohol-cue reactivity as a neural risk marker for longer-term alcohol-related outcomes.

A second strength is the relatively high overall model performance, especially given the modest sample size. The predictive accuracy, classification, sensitivity, and specificity were all similar to that seen in our original 3-factor PET model (17). Although the current finding will need replication, it is the third test of our model, and each has proven to be statistically robust in sample sizes ranging from 44 to 1338 (17,18).

Despite these strengths, several limitations should be considered. First, as noted, the current sample size is modest. Replication will be needed, but the sample size is within the norms of much fMRI research, and the hypotheses followed directly from our previous 2 studies (17,18). Moreover, our bootstrapping analyses supported an association between higher CTQ scores, higher EXT behavior scores, and stronger mesocorticolimbic responses to alcohol versus juice cues and later alcohol use problems, and this pattern remained when sex was added as a fourth factor. Across models, sex independently predicted the outcome, with women showing a lower probability of alcohol use problems. In comparison, out-of-sample validation (e.g., k-fold cross-validation) was not implemented due to the limited sample size; thus, the reported classification accuracy and AUC values should be interpreted as in-sample estimates. Future studies with larger cohorts are needed to validate these models using independent samples. Second, in the current study, childhood trauma was not a significant predictor of later alcohol use problems in the primary high versus low AUDIT models. This may reflect an effect of early-life trauma on broad transdiagnostic risk rather than uniquely increasing vulnerability to alcohol use (3). Alternatively, it may reflect an issue of statistical power. The inclusion of CTQ scores strengthened the model (relative to BOLD and EXT behavior alone), and in the secondary analyses using continuous follow-up AUDIT scores, CTQ scores emerged as an independent predictor across models. Moreover, there is evidence that early-life adversity has various effects on adult mesocorticolimbic reactivity to emotionally relevant stimuli (64) including augmented responses to drug-related cues in people with a current addiction (65).

Third, in our sample, the high AUDIT group was defined as ≥5, below the clinical cutoff of 8. Although this group’s mean score of 8.2 ± 2.9 confirmed that at least some participants had clinically relevant alcohol use problems, the current results are more cautiously interpreted as identifying those who will develop higher AUDIT scores rather than alcohol use disorders.

Fourth, participants were selected from the upper and lower 30% of the EXT score distribution to maximize group contrast, which enhances sensitivity to individual differences and improves statistical power in a modest sample. However, this approach excludes the middle 40% of the distribution, limiting generalizability to individuals with more moderate externalizing traits.

Fifth, mediation and moderation effects were both identified in our previous fMRI study with a large sample (18). That is, the effect of high CTQ scores on clinical outcomes was partially mediated by higher EXT behaviors and moderated by fMRI BOLD responses (18). Future studies with larger samples will be needed to test whether these effects on transdiagnostic risk also influence more specific outcomes.

### Conclusions

The current study, along with our previous findings (17,18), tentatively identifies a hierarchical diathesis-stress model with both transdiagnostic and precision psychiatry features. High mesocorticolimbic reactivity to distinct environmental cues may shape an overarching general liability into a more specific clinical outcome.

## Supplementary Material

Supplemental Methods and Tables S1–S24

Key Resources Table
