# Supplementary material for: Reimagining the Biopsychosocial Model: Transdiagnostic Factors to Precision Psychiatry
Source: Biol Psychiatry Glob Open Sci. 2026 Apr 17;6(4):100734. doi: 10.1016/j.bpsgos.2026.100734 (PMC13254389; doi:10.1016/j.bpsgos.2026.100734)
Supplement: Supplemental Methods and Tables S1–S24 [file mmc1.pdf]

SUPPLEMENTARY INFORMATION

Reimagining the Biopsychosocial Model: Transdiagnostic Factors to Precision Psychiatry

Nikolic *et al.*

Table S1: Three-factor binomial regression models with each ROI for the Alcohol vs Juice contrast predict high vs low alcohol use at follow-up: including positive predictive value, negative predictive value, B (S.E), and Wald.

**Alcohol vs Juice BOLD** (p-value, OR, 95% CI), B (S.E), Wald	**EXT** (p-value, OR, 95% CI), B (S.E), Wald	**CTQ** (p-value, OR, 95% CI), B (S.E), Wald	**Full model** (χ^2 , p-value) Nagelkerke R^2	AUC(CI), **classification predictive accuracy, sensitivity & specificity**	**PPV and NPV**
PCC (p=0.007, 5.55, 1.613-19.112), 1.714 (0.631), 7.390	p=0.018, 3.09, 1.21-7.92, 1.130 (0.479), 5.572	p=0.42, 1.43, 0.59-3.43, 0.360 (0.445), 0.653	(16.6, p<0.001), 0.42	0.821 (0.698-0.944), 75%, 73.7% and 76%	70.0% and 79.2%
AS (p=0.036, 3.377, 1.085-10.507), 1.217 (0.579), 4.415	p=0.026, 2.62, 1.13-6.08, 0.961 (0.430), 4.988	p=0.35, 1.57, 0.61-4.02, 0.451 (0.479), 0.883	(12.84, p=0.005), 0.34	0.798 (0.669-0.926), 63.6%, 52.6% and 72%	58.82% and 66.67%
vmPFC (p=0.039, 2.756, 1.053-7.213), 1.014 (0.491), 4.268	p=0.031, 2.55, 1.09-5.98, 0.938 (0.434), 4.673	p=0.27, 1.64, 0.68-3.97, 0.494 (0.451), 1.196	(12.09, p=0.007), 0.32	0.785 (0.648-0.922), 70.5%, 57.9% and 80%	68.75% and 71.43%
VS (p=0.051, 3.267, 0.996-10.718), 1.184 (0.606), 3.813	p=0.035, 2.47, 1.07-5.71, 0.903 (0.428), 4.464	p=0.287, 1.65, 0.66-4.13, 0.499 (0.469), 1.134	(11.82, p=0.008), 0.32	0.789 (0.658-0.921), 63.6%, 47.4% and 76%	60.0% and 65.52%
SS (p=0.157, 1.861, 0.787-4.398), 0.621 (0.439), 2.002	p=0.039, 2.355, 1.043-5.315, 0.857 (0.415), 4.254	p=0.330, 1.539, 0.647-3.662, 0.431(0.442), 0.950	(8.636, p=0.035), 0.239	0.733(0.582-0.883), 68.2%, 52.6% and 80%	66.7% and 69%
Amygdala (p=0.205, 1.747, 0.737-	p=0.043, 2.223, 1.024-	p=0.331, 1.566, 0.634-	(8.154, p=0.043), 0.227	0.728 (0.575-0.882), 65.9%,	66.7% and 65.6%

4.143), 0.558 (0.440), 1.606	4.826, 0.799 (0.395), 4.084	3.871, 0.449 (0.462), 0.945		42.1% and 84%	
SN/VTA (p=0.262, 1.541, 0.723-3.281),0.432 (0.386), 1.256	p=0.045, 2.193, 1.019-4.717, 0.785 (0.391), 4.036	p=0.373, 1.520, 0.605-3.820, 0.419 (0.470), 0.794	(7.720, p=0.052), 0.216	0.716 (0.556-0.875), 65.9%, 47.4% and 80%	64.3% and 66.7%
dlPFC (p=0.074, 2.482, 0.916-6.726), 0.909 (0.509), 3.193	p=0.038, 2.277, 1.047-4.954, 0.823 (0.397), 4.306	p=0.369, 1.514, 0.612-3.745, 0.415 (0.462), 0.807	(11.031, p=0.012), 0.298	0.777 (0.640-0.913), 68.2%, 57.9% and 76%	64.7% and 70.4%
ACC (p=0.069, 2.635, 0.927-7.488), 0.969 (0.533), 3.306	p=0.027, 2.589, 1.117-6.004, 0.951 (0.429), 4.917	p=0.340, 1.558, 0.626-3.875, 0.443 (0.465), 0.910	(11.021, p=0.012), 0.297	0.768 (0.631-0.906), 63.6%, 52.6% and 72%	58.8% and 66.7%
Insula (p=0.422, 1.398, 0.617-3.168), 0.335 (0.417), 0.644	p=0.045, 2.201, 1.018-4.760, 0.789 (0.394), 4.019	p=0.301, 1.579, 0.664-3.752, 0.457 (0.442), 1.070	(7.083, p=0.069), 0.200	0.712 (0.554-0.869), 70.5%, 57.9% and 80%	68.8% and 71.4%

Table S2: Three factor model: Alcohol vs Juice ROI composite, EXT scores and CTQ scores predict high vs low alcohol use at follow-up

Alcohol vs. Juice composite BOLD (p-value, OR, 95% CI)	EXT (p-value, OR, 95% CI)	CTQ (p-value, OR, 95% CI)	Full model (χ^2, p-value), Nagelkerke R^2	AUC (CI), classification predictive accuracy, sensitivity & specificity
p = .043, 3.946, 1.041–14.949	p = .026, 2.670, 1.124–6.341	p = .313, 1.617, 0.635–4.116	(12.166, p = .007), 0.324	AUC (CI): not shown in this output; 68.2%, 52.6% and 80.0%

Table S3: Likelihood Ratio Tests Comparing Three-Factor Models (CTQ + EXT + ROI-Specific BOLD Response Alcohol vs Juice) to the Two-Factor Model (EXT + BOLD Response Alcohol vs Juice) for predicting follow-up alcohol use. -2LL= -2 log likelihood, Δ -2 LL= difference in -2 Log Likelihood values between the two models.

**ROI**	**Model 1** (EXT+fMRI BOLD) -2 LL	**Model 2** (EXT+fMRI BOLD+CTQ) -2 LL	**Δ -2 LL** (χ^2)	**df** (Δ Parameters)	**p-value** (Model 1 vs Model 2)
PCC	45.126	43.573	1.553	1	0.213
AS	52.392	47.332	5.06	1	0.025
vmPFC	52.093	48.085	4.008	1	0.045
VS	53.381	48.355	5.026	1	0.025
SS	55.564	51.540	4.024	1	0.0449
Amygdala	56.615	52.022	4.593	1	0.0321
SN/VTA	57.017	52.456	4.561	1	0.0327
dlPFC	53.824	49.145	4.679	1	0.0305
ACC	53.434	49.155	4.279	1	0.0386
Insula	57.062	53.093	3.969	1	0.0463

Table S4: Likelihood Ratio Tests Comparing Three-Factor Models (CTQ + EXT + Alcohol vs Juice ROI composite) to the Two-Factor Model (EXT + Alcohol vs Juice ROI composite) for predicting follow-up alcohol use.

**Model 1** (EXT+ Alcohol vs Juice ROI composite) -2 LL	**Model 2** (EXT+ Alcohol vs Juice ROI composite +CTQ) -2 LL	**Δ -2 LL** (χ^2)	**df** (Δ Parameters)	**p-value** (Model 1 vs Model 2)
52.276	48.010	4.266	1	.039

Table S5: Four-factor model: Alcohol vs. Juice BOLD signal, CTQ scores, EXT scores, and AUDIT scores at age 18 predict high vs low alcohol use at follow-up.

**ROI**	**Alcohol vs Juice BOLD** (p-value, OR, 95% CI)	**EXT** (p-value, OR, 95% CI)	**CTQ** (p-value, OR, 95% CI)	**AUDIT age 18** (p-value, OR, 95% CI)	**Full model** (χ^2, p-value), Nagelkerke R^2	**AUC (CI), classification predictive accuracy, sensitivity & specificity**
PCC	p=0.009, 5.34, 1.53–18.68	p=0.028, 2.95, 1.12–7.72	p=0.475, 1.38, 0.56–3.37	p=0.711, 1.20, 0.45–3.20	(16.75, p=0.002), 0.425	0.827 (0.707-0.948), 75%, 73.7% and 76.0%
AS	p=0.048, 3.22, 1.01–10.22	p=0.043, 2.43, 1.03–5.77	p=0.419, 1.48, 0.57–3.83	p=0.564, 1.32, 0.52–3.38	(13.19, p=0.010), 0.348	0.789 (0.659-0.920), 68.2%, 52.6% and 80.0%
vmPFC	p=0.048, 2.62, 1.01–6.82	p=0.057, 2.32, 0.98–5.50	p=0.401, 1.48, 0.59–3.69	p=0.431, 1.43, 0.59–3.44	(12.75, p=0.013), 0.337	0.792 (0.657-0.927), 70.5%, 63.2% and 76.0%
VS	p=0.065, 3.08, 0.93–10.20	p=0.064, 2.25, 0.96–5.28	p=0.377, 1.52, 0.60–3.87	p=0.465, 1.40, 0.57–3.41	(12.38, p=0.015), 0.329	0.798 (0.669-0.927), 63.6%, 47.4% and 76.0%
ACC	p=0.090, 2.47, 0.87–6.99	p=0.049, 2.36, 1.00–5.56	p=0.463, 1.42, 0.56–3.61	p=0.463, 1.39, 0.58–3.35	(11.593, p=0.021), 0.311	0.777 (0.642-0.911), 68.2%, 52.6% and 80%
SS	p=0.247, 1.69, 0.70–4.08	p=0.073, 2.15, 0.93–4.95	p=0.447, 1.42, 0.58–3.48	p=0.519, 1.35, 0.54–3.38	(9.07, p=0.059), 0.250	0.752 (0.607-0.896) 70.5%, 52.6% and 84.0%
Amygdala	p=0.256, 1.64, 0.70–3.84	p=0.080, 2.02, 0.92–4.42	p=0.486, 1.39, 0.55–3.50	p=0.379, 1.49, 0.62–3.58	(8.99, p=0.061), 0.248	0.745 (0.597-0.894), 65.9%, 42.1% and 84.0%
SN/VTA	p=0.332, 1.46, 0.68–3.16	p=0.081, 2.00, 0.92–4.36	p=0.529, 1.35, 0.53–3.46	p=0.373, 1.48, 0.62–3.53	(8.571, p=0.073), 0.237	0.741 (0.595-0.887), 68.2%,

						47.4% and 84.0%
dlPFC	p=0.106, 2.30, 0.84–6.34	p=0.062, 2.14, 0.96–4.76	p=0.472, 1.41, 0.55–3.57	p=0.594, 1.29, 0.51–3.26	(11.33, p=0.023), 0.304	0.787 (0.654-0.921), 70.5%, 52.6% and 84%
Insula	p = 0.590, 1.26, 0.55–2.90	p = 0.087, 1.98, 0.91–4.34	p = 0.492, 1.38, 0.55–3.41	p = 0.381, 1.47, 0.62–3.50	(7.90, p = 0.095), 0.22	0.731 (0.579-0.882), 72.7%, 57.9%, 84.0%

Table S6: Four-factor model: Alcohol vs Juice ROI composite, CTQ scores, EXT scores, and AUDIT scores at age 18 predict high vs low alcohol use at follow-up.

**Alcohol vs Juice ROI composite** (p-value, OR, 95% CI)	**EXT** (p-value, OR, 95% CI)	**CTQ** (p-value, OR, 95% CI)	**AUDIT age 18** (p-value, OR, 95% CI)	**Full model** (χ^2, p-value), Nagelkerke R^2	**AUC (CI), classification predictive accuracy, sensitivity & specificity**
p=0.065, 3.60, 0.92–14.02	p=0.047, 2.49, 1.01–6.14	p=0.389, 1.53, 0.58–3.98	p=0.664, 1.23, 0.48–3.18	(12.36, p=0.015), 0.329	0.783 (0.650–0.916), 70.5%, 52.6% and 84.0%

Table S7: Likelihood Ratio Tests (LRT) Comparing Four-Factor Models (CTQ + EXT + ROI-Specific BOLD Response Alcohol vs Juice + Sex) to Three-Factor Models (CTQ + EXT + ROI-Specific BOLD Response Alcohol vs Juice) for predicting follow-up alcohol use. -2LL= -2 log likelihood, Δ -2 LL= difference in -2 Log Likelihood values between the two models.

**ROI**	**Model 1** (EXT + fMRI BOLD + CTQ) -2 LL	**Model 2** (EXT + fMRI BOLD + CTQ+Sex) -2 LL	**Δ -2 LL** (χ^2)	**df** (Δ Parameters)	**p-value** (Model 1 vs Model 2)
PCC	43.573	39.181	4.392	1	0.0361
AS	47.332	40.970	6.362	1	0.0117
vmPFC	48.085	43.534	4.551	1	0.0329
VS	48.355	42.628	5.727	1	0.0167
SS	51.540	46.504	5.036	1	0.025
Amygdala	52.022	47.352	4.670	1	0.031
dlPFC	49.145	46.062	3.083	1	0.079
SN/VTA	52.405	46.974	5.431	1	0.019
ACC	49.155	43.041	6.114	1	0.013
Insula	53.093	49.235	3.853	1	0.050

Table S8: Three-factor linear regression model (alcohol vs. juice BOLD, EXT, CTQ) predicts continuous follow-up AUDIT scores.

ROI	Alcohol vs. Juice BOLD (B (SE), β, t, p)	EXT (B (SE), β, t, p)	CTQ (B (SE), β, t, p)	Full model (F(df1,df2), p)	R^2
PCC	1.513 (0.610), .323, 2.480, .017	1.088 (0.502), .291, 2.166, .036	1.341 (0.545), .329, 2.459, .018	F(3,40) = 6.605, p < .001	.331
AS	0.916 (0.573), .217, 1.598, .118	1.065 (0.525), .284, 2.028, .049	1.417 (0.567), .347, 2.500, .017	F(3,40) = 5.051, p = .005	0.275
SS	0.509 (0.575), .123, 0.885, .382	1.008 (0.535), .269, 1.882, .067	1.458 (0.580), .357, 2.512, .016	F(3,40) = 4.286, p = .010	.243
VS	0.873 (0.619), .192, 1.410, .166	1.010 (0.525), .270, 1.922, .062	1.463 (0.571), .359, 2.561, .014	F(3,40) = 4.806, p = .006	.265
Amygdala	0.559 (0.597), .130, 0.936, .355	0.996 (0.533), .266, 1.868, .069	1.465 (0.580), .359, 2.526, .016	F(3,40) = 4.326, p = .010	.245
SN/VTA	0.700 (0.543), .177, 1.290, .205	1.022 (0.529), .273, 1.933, .060	1.409 (0.573), .345, 2.459, .018	F(3,40) = 4.666, p = .007	.259
vmPFC	0.634 (0.531), .164, 1.193, .240	1.011 (0.530), .270, 1.909, .063	1.457 (0.575), .357, 2.533, .015	F(3,40) = 4.563, p = .008	.255
dlPFC	0.730 (0.515), .193, 1.418, .164	1.010 (0.525), .270, 1.923, .062	1.406 (0.570), .345, 2.465, .018	F(3,40) = 4.816, p = .006	.265
ACC	0.761 (0.553), .190, 1.375, .177	1.077 (0.532), .288, 2.022, .050	1.442 (0.571), .353, 2.524, .016	F(3,40) = 4.765, p = .006	.263
Insula	0.267 (0.605), .064, 0.441, .662	0.991 (0.544), .265, 1.821, .076	1.468 (0.593), .360, 2.476, .018	F(3,40) = 4.032, p = .013	.232

Table S9: F-change tests comparing three-factor models (CTQ + EXT + ROI-Specific Alcohol vs. Juice BOLD Response) to two-factor models (CTQ + EXT) for predicting continuous follow-up AUDIT scores. ΔR^2 = change in R^2 between models; Fchange tests whether the addition of the ROI term significantly improves model fit.

ROI	Model 1 R^2 (EXT+CTQ)	Model 2 R^2 (EXT+CTQ+ROI)	ΔR^2	Fchange (1,40)	p (ΔR^2)
PCC	.228	.331	.103	6.151	**.017**
AS	.228	.275	.046	2.553	.118
VS	.228	.265	.037	1.987	.166
ACC	.228	.263	.035	1.891	.177
dlPFC	.228	.265	.037	2.011	.164
vmPFC	.228	.255	.027	1.424	.240
SN/VTA	.228	.259	.031	1.663	.205
Amygdala	.228	.245	.017	0.876	.355
SS	.228	.243	.015	0.782	.382
Insula	.228	.232	.004	0.194	.662

Table S10: F-change tests comparing four-factor models (EXT + CTQ + ROI BOLD + Sex) to three-factor models (EXT + CTQ + ROI BOLD) for predicting continuous follow-up AUDIT scores. ΔR^2 = change in R^2 between models; Fchange tests whether the addition of the ROI term significantly improves model fit.

ROI	Model 1 R^2 (EXT+CTQ+ROI)	Model 2 R^2 (EXT+CTQ+ROI +Sex)	ΔR^2	Fchange (df1, df2)	p
PCC	.331	.341	.010	0.597 (1,39)	.444

Table S11: Three-factor linear regression model (Alcohol vs Juice ROI composite, EXT, CTQ) predicts continuous follow-up AUDIT scores.

Alcohol vs. Juice ROI composite (B (SE), β, t, p)	EXT (B (SE), β, t, p)	CTQ (B (SE), β, t, p)	Full model (F(df1,df2), p)	R^2
1.110 (0.696), .218, 1.594, .119	1.072 (0.526), .286, 2.039, .048	1.462 (0.567), .358, 2.577, .014	F(3,40) = 5.046, p = .005	0.275

Table S12: F-change tests comparing three-factor model (EXT + CTQ + Alcohol vs Juice ROI composite) to two-factor model (EXT + CTQ) for predicting continuous follow-up AUDIT scores. ΔR^2 = change in R^2 between models; Fchange tests whether the addition of the ROI term significantly improves model fit.

Model 1 R^2 (EXT+CTQ)	Model 2 R^2 (EXT+CTQ+ Alcohol vs Juice ROI composite)	ΔR^2	Fchange (df1, df2)	p
.228	.275	.046	2.542 (1,40)	.119

Table S13: EXT scores predict DSM-5 disorders seven years later.

Predictor	p-value, OR, 95% CI	Full model (χ^2, p-value) Nagelkerke R^2	AUC (CI), classification predictive accuracy, sensitivity & specificity
**EXT scores**	p=0.050, 2.059, 0.99-4.25	(4.27, p= 0.039), 0.124	0.695 (0.540-0.849), 63.6%, 68% and 57.9%

Table S14: Likelihood Ratio Tests Comparing 2-Factor Model (EXT+CTQ) to 1-factor model (EXT) for predicting DSM-5 disorders seven years later. -2LL= -2 log likelihood, Δ -2 LL= difference in -2 Log Likelihood values between the two models.

**Model 1** (EXT) -2 LL	**Model 2** (EXT+ CTQ) -2 LL	**Δ -2 LL** (χ^2)	**df** (Δ Parameters)	**p-value** (Model 1 vs Model 2)
55.909	53.277	2.632	1	0.1047

Table S15: 2-factor Model (EXT+CTQ) predicts DSM-5 disorders seven years later.

**EXT scores** (p-value, OR, 95% CI)	**CTQ** (p-value, OR, 95% CI)	**Full model** (χ^2, p-value) Nagelkerke R^2	**AUC (CI), classification predictive accuracy**, sensitivity & specificity
p=0.026, 2.531, 1.118-5.734)	p=0.127, 0.528, 0.23-1.2	(6.9, 0.032), 0.195	0.604 (0.432-0.776), 68.2%, 76% and 57.9%

Table S16: Likelihood Ratio Tests Comparing Three-Factor Models (CTQ + EXT + ROI-Specific BOLD Response Alcohol vs Juice) to the One-Factor Model (EXT response) for predicting DSM-5 disorders. -2LL= -2 log likelihood, Δ -2 LL= difference in -2 Log Likelihood values between the two models.

**ROI**	**Model 1** (EXT) -2 LL	**Model 2** (EXT + fMRI BOLD + CTQ) -2 LL	**Δ -2 LL** (χ^2)	**df** (Δ Parameters)	**p-value** (Model 1 vs Model 2)
PCC	55.909	52.053	3.856	2	0.145
AS	55.909	52.771	3.138	2	0.208
vmPFC	55.909	53.235	2.674	2	0.263
SS	55.909	53.221	2.688	2	0.261
VS	55.909	52.971	2.938	2	0.230
Amygdala	55.909	53.218	2.691	2	0.260
SN/VTA	55.909	53.060	2.849	2	0.241
dlPFC	55.909	53.169	2.740	2	0.254
ACC	55.909	52.408	3.501	2	0.174
Insula	55.909	53.159	2.750	2	0.253

Table S17: Binomial regression three-factor model: Alcohol vs. Juice BOLD signal, CTQ scores, and EXT scores predict future DSM-5 disorders.

**Alcohol vs Juice BOLD** (p-value, OR, 95% CI)	**EXT** (p-value, OR, 95% CI)	**CTQ** (p-value, OR, 95% CI)	**Full model** (χ^2, p-value) Nagelkerke R^2	**AUC (CI), classification predictive accuracy, sensitivity & specificity**
PCC (p=0.281, 1.655, 0.662-4.134)	p=0.020, 2.754, 1.175-6.454	p=0.113, 0.491, 0.204-1.184	(8.123, p=0.044), 0.226	0.701 (0.542-0.860), 61.4%, 72% and 47.4%
ACC (p=0.383, 0.655, 0.253-1.693)	p=0.040, 2.358, 1.042-5.338	p=0.125, 0.529, 0.234-1.194	(7.769, p=0.051), 0.217	0.539 (0.362-0.716), 72.7%, 80.0% and 63.2%
AS (p=0.492, 0.733, 0.302-1.778)	p=0.033, 2.442, 1.073-5.556	p=0.131, 0.531, 0.234-1.207	(7.405, p=0.060), 0.208	0.549 (0.372-0.727), 70.5%, 80.0% and 57.9%
VS (p=0.591, 0.768, 0.294-2.008)	p=0.030, 2.474, 1.093-5.598	p=0.122, 0.526, 0.233-1.187	(7.205, p=0.066), 0.203	0.566 (0.391-0.741), 70.5%, 76.0% and 63.2%
SS (p=0.815, 1.104, 0.482-2.532)	p=0.028, 2.59, 1.11-6.04	p=0.13, 0.53, 0.23-1.21	(6.95, p=0.073), 0.196	0.619 (0.449-0.789), 63.6%, 68% and 57.9%
Amygdala (p=0.81, 1.107, 0.484-2.53)	p=0.027, 2.57, 1.12-5.95	p=0.13, 0.53, 0.23-1.21	(6.96, p=0.073), 0.196	0.619 (0.448-0.790), 65.9%, 72% and 57.9%
SN/VTA (p=0.64, 0.834, 0.387-1.798)	p=0.031, 2.46, 1.08-5.57	p=0.14, 0.53, 0.23-1.22	(7.12, p=0.068), 0.2	0.575 (0.398-0.752), 70.5%, 76% and 63.2%
vmPFC (p=0.84, 0.926, 0.441-1.94)	p=0.030, 2.49, 1.09-5.69	p=0.13, 0.53, 0.23-1.19	(6.94, p=0.074), 0.196	0.579 (0.406-0.752), 70.5%, 76% and 63.2%
dlPFC (p=0.75, 0.88, 0.41-1.88)	p=0.028, 2.50, 1.1-5.67	p=0.13, 0.53, 0.23-1.21	(7.01, p=0.072), 0.198	0.573 (0.399-0.747), 70.5%, 76% and 63.2%
Insula (p=0.73, 1.16, 0.49-2.75)	p=0.028, 2.65, 1.1-6.31	p=0.14, 0.53, 0.23-1.23	(7.02, p=0.071), 0.198	0.611 (0.439-0.782), 68.2%, 72% and 63.2%

Table S18: Three-factor binomial regression models with each ROI for the Alcohol vs Water contrast predict follow-up high vs low alcohol use.

**Alcohol vs Water BOLD** (p-value, OR, 95% CI)	**EXT** (p-value, OR, 95% CI)	**CTQ** (p-value, OR, 95% CI)	**Full model (χ^2, p-value)** Nagelkerke R^2	**AUC (CI), classification predictive accuracy, sensitivity & specificity**
PCC (p=0.233, 1.65, 0.73-3.74)	p=0.066, 2.02, 0.953-4.279	p=0.528, 1.33, 0.55-3.26	(7.918, p=0.048), 0.221	0.724 (0.567-0.882), 70.5%, 63.2% and 76.0%
AS (p=0.236, 1.59, 0.74-3.43)	p=0.042, 2.24, 1.03-4.85	p=0.504, 1.36, 0.554-3.325	(7.898, p=0.048), 0.220	0.724 (0.563-0.885), 70.5%, 57.9% and 80%
vmPFC (p=0.232, 1.54, 0.76-3.13)	p=0.051, 2.14, 0.99-4.57	p=0.395, 1.45, 0.61-3.43	(7.96, p=0.047), 0.22	0.739 (0.578-0.899), 77.3%, 68.4% and 84%
VS (p=0.224, 1.64, 0.74-3.61)	p=0.052, 2.12, 0.99-4.53	p=0.488, 1.37, 0.56-3.3	(8.020, p=0.046), 0.224	0.735 (0.573-0.897), 70.5%, 57.9% and 80%
SS (p=0.517, 1.26, 0.62-2.56)	p=0.051, 2.12, 0.99-4.46	p=0.45, 1.40, 0.58-3.38	(6.84, p=0.077), 0.193	0.699 (0.533-0.865), 63.6%, 52.6% and 72.0%
Amygdala (p=0.97, 1.01, 0.51-1.99)	p=0.058, 2.05, 0.98-4.31	p=0.36, 1.49, 0.63-3.57	(6.42, p=0.093), 0.182	0.701 (0.535-0.867), 63.6%, 52.6% and 72.0%
SN/VTA (p=0.37, 1.39, 0.68-2.89)	p=0.045, 2.24, 1.02-4.91	p=0.50, 1.37, 0.55-3.47	(7.25, p=0.064), 0.204	0.709 (0.550-0.868), 63.6%, 47.4% and 76.0%
dlPFC (p=0.29, 1.48, 0.72-3.04)	p=0.043, 2.20, 1.03-4.72	p=0.41, 1.43, 0.61-3.39	(7.66, p=0.054), 0.214	0.712 (0.545-0.878), 70.5%, 63.2% ad 76.0%
ACC (p=0.39, 1.37, 0.67-2.77)	p=0.045, 2.17, 1.02-4.62	p=0.39, 1.46, 0.61-3.48	(7.19, p=0.066), 0.202	0.728 (0.567-0.890), 72.7%, 63.2% and 80.0%
Insula (p=0.54, 1.26, 0.60-2.64)	p=0.048, 2.14, 1.01-4.55	p=0.39, 1.45, 0.62-3.41	(6.79, p=0.079), 0.192	0.695 (0.525-0.864), 63.6%, 52.6% and 72.0%

Three factor model (EXT+ CTQ + Alcohol vs Water contrast ROI) predicts higher vs lower AUDIT group at follow-up.

The primary fMRI measure contrasted responses to the alcohol plus juice cues vs juice alone (i.e., controlling for appetitive effects of juice). This noted, exploratory analyses contrasting the cues for alcohol vs. water yielded similar results, such that higher future AUDIT scores were predicted by the combination of high CTQ scores, high EXT scores, and high Alc vs. Water contrast responses in the VS, AS, vmPFC, and PCC models ($p<0.048$, Tables S18). The addition of ROI responses in Alcohol vs Water and CTQ did not improve model fit compared to EXT alone (all $p>0.275$, Table S19), but adding sex as a fourth factor improved model fit compared to three-factor models incorporating the VS, AS, and vmPFC ($p<0.056$) (Tables S20, S21).

Table S19: Likelihood Ratio Tests Comparing Three-Factor Models (CTQ + EXT + ROI-Specific BOLD Response Alcohol vs Water) to the One-Factor Model (EXT response) for predicting follow-up alcohol use. -2LL= -2 log likelihood, Δ -2 LL= difference in -2 Log Likelihood values between the two models.

**ROI**	**Model 1** (EXT) -2 LL	**Model 2** (EXT + fMRI BOLD + CTQ) -2 LL	**Δ -2 LL** (χ^2)	**df** (Δ Parameters)	**p-value** (Model 1 vs Model 2)
PCC	54.739	52.258	2.481	2	0.289
AS	54.739	52.278	2.461	2	0.292
vmPFC	54.739	52.218	2.521	2	0.284
VS	54.739	52.156	2.583	2	0.275
SS	54.739	53.335	1.404	2	0.496
Amygdala	54.739	53.756	0.983	2	0.612
SN/VTA	54.739	52.930	1.809	2	0.405
dlPFC	54.739	52.515	2.224	2	0.329
ACC	54.739	52.987	1.752	2	0.417
Insula	54.739	53.387	1.352	2	0.509

Table S20: Likelihood Ratio Tests Comparing Four-Factor Models (CTQ + EXT + ROI-Specific BOLD Response Alcohol vs Water + Sex) to the Three-Factor Model (CTQ + EXT +

ROI-Specific BOLD Response Alcohol vs Water) for predicting follow-up alcohol use. -2LL= -2 log likelihood, Δ -2 LL= difference in -2 Log Likelihood values between the two models.

**ROI**	**Model 1** (EXT + fMRI BOLD + CTQ) -2 LL	**Model 2** (EXT + fMRI BOLD + CTQ+Sex) -2 LL	**Δ -2 LL** (χ^2)	**df** (Δ Parameters)	**p-value** (Model 1 vs Model 2)
PCC	52.258	48.877	3.381	1	0.0659
AS	52.278	48.127	4.151	1	0.0416
vmPFC	52.218	48.567	3.651	1	0.0560
VS	52.156	48.365	3.791	1	0.0515

Table S21: Binomial regression models with the Alcohol vs Water contrast (fMRI BOLD + EXT +CTQ + Sex) for predicting follow-up high vs low alcohol use.

**Alcohol vs Water BOLD** (p-value, OR, 95% CI)	**EXT** (p-value, OR, 95% CI)	**CTQ** (p-value, OR, 95% CI)	**Sex** (p-value, OR, 95% CI)	**Full model** (χ^2 , p-value), Nagelkerke R^2	**AUC (CI), classification predictive accuracy, sensitivity & specificity**
PCC (p=0.232, 1.717, 0.708-4.166)	p=0.060, 2.130, 0.967-4.690	p=0.701, 1.184, 0.501-2.797	p=0.073, 0.278, 0.069-1.126	(11.299, p=0.023), 0.304	0.781 (0.635-0.927), 77.3% 73.7% and 80%
AS (p=0.155, 1.85, 0.79-4.32)	p=0.041, 2.305, 1.04-5.14	p=0.750, 1.15, 0.484-2.737	p=0.050, 0.232, 0.054-0.998	(12.049, p=0.017), 0.321	0.787 (0.637-0.938), 79.5%, 73.7% and 84%
vmPFC (p=0.205, 1.650, 0.761-3.579)	p=0.056, 2.142, 0.980-4.683	p=0.509, 1.321, 0.577-3.025	p=0.063, 0.263, 0.064-1.073	(11.609, p=0.021), 0.311	0.781 (0.629-0.933), 81.8%, 73.7% and 88%
VS (p=0.184, 1.809, 0.754-4.344)	p=0.057, 2.144, 0.976-4.711	p=0.656, 1.213, 0.52-2.828	P=0.059, 0.252, 0.060-1.053	(11.812, p=0.019), 0.316	0.785 (0.634-0.936), 79.5%, 68.4% and 88%
ACC (p=0.304, 1.520, 0.685-3.372)	p=0.043, 2.249, 1.028-4.922	p=0.534, 1.301, 0.567-2.986	p=0.059, 0.256, 0.062-1.056	(10.960, p=0.027), 0.296	0.781 (0.628-0.934), 81.8% 73.7% and 88%
Amygdala (p=0.57, 1.23, 0.59-2.53)	p=0.046, 2.26, 1.01-5.04	p=0.60, 1.26, 0.53-2.99	p=0.063, 0.25, 0.060-1.07	(10.09, p=0.039), 0.28	0.779 (0.622-0.936), 79.5%, 68.4% and 88.0%
SN/VTA (p=0.19, 1.68, 0.77-3.69)	p=0.037, 2.53, 1.06-6.05	p=0.80, 1.13, 0.45-2.84	p=0.046, 0.23, 0.053-0.97	(11.53, p=0.021), 0.31	0.796 (0.648-0.944), 84.1%, 73.7% ad 92.0%
SS (p=0.28, 1.57, 0.69-3.53)	p=0.047, 2.29, 1.01-5.18	p=0.72, 1.17, 0.49-2.78	p=0.051, 0.23, 0.053-1.0	(11.0, p=0.027), 0.29	0.794 (0.640-0.948), 79.5%, 63.2% and 92.0%
dlPFC (p=0.28, 1.57, 0.69-3.56)	p=0.042, 2.27, 1.03-4.98	p=0.55, 1.29, 0.56-2.94	p=0.067, 0.27, 0.066-1.09	(11.19, p=0.025), 0.30	0.787 (0.630-0.945), 86.4%, 78.9% and 92.0%

Insula (p=0.44, 1.38, 0.61-3.13)	p=0.044, 2.27, 1.02-5.04	p=0.57, 1.27, 0.56-2.91	p=0.065, 0.27, 0.066-1.083	(10.39, p=0.034), 0.28	0.781 (0.620-0.942), 86.4%, 78.9% and 92.0%

Table S22: Single-factor binomial regression models predict follow-up high vs low alcohol use: EXT scores and posterior cingulate cortex Alcohol vs Juice BOLD Response**.**

Predictor	p-value, OR, 95% CI	Full model (χ^2, p-value) Nagelkerke R^2	AUC (CI), classification predictive accuracy, sensitivity & specificity
EXT scores	p=0.028, 2.242, 1.093-4.599	(5.438, p=0.020), 0.156	0.695 (0.540-0.849), 65.9%, 63.2% and 68.0%
Alcohol vs Juice PCC BOLD	p=0.015, 3.41, 1.274-9.122	(7.232, p=0.007), 0.203	0.724 (0.572-0.877), 65.9%, 57.9% and 72.0%

Table S23: Bootstrapped three-factor model: alcohol vs juice BOLD signal, EXT scores, and CTQ scores predict follow-up high vs low alcohol use.

ROI	Alcohol vs Juice BOLD: p, OR [95% CI], B (SE)	EXT: p, OR [95% CI], B (SE)	CTQ: p, OR [95% CI], B (SE)
PCC	p = .002, 5.55 [2.28, 59.17], 1.714 (0.858)	p = .003, 3.10 [1.54, 17.70], 1.130 (0.616)	p = .405, 1.43 [0.19, 4.26], 0.360 (0.780)
AS	p = .008, 3.38 [1.60, 17.88], 1.217 (0.652)	p = .012, 2.61 [1.20, 9.59], 0.961 (0.549)	p = .245, 1.57 [0.50, 6.39], 0.451 (0.646)
vmPFC	p = .027, 2.756 [1.116, 26.08], 1.014 (0.804)	p = .022, 2.555 [1.123, 14.23], 0.938 (0.636)	p = .186, 1.638 [0.409, 5.14], 0.494 (0.695)
VS	p = .018, 3.27 [1.35, 25.77], 1.184 (0.757)	p = .027, 2.47 [0.95, 9.25], 0.903 (0.572)	p = .198, 1.65 [0.54, 6.24], 0.499 (0.638)
ACC	p = .037, 2.64 [1.10, 15.78], 0.969 (0.620)	p = .014, 2.59 [1.15, 9.24], 0.951 (0.539)	p = .249, 1.56 [0.48, 6.53], 0.443 (0.628)
SS	p = .206, 1.86 [0.78, 10.50], 0.621 (0.680)	p = .016, 2.36 [1.18, 9.05], 0.857 (0.505)	p = .258, 1.54 [0.43, 5.19], 0.431 (0.593)
Amygdala	p = .124, 1.75 [0.74, 4.69], 0.558 (0.457)	p = .019, 2.22 [1.03, 6.07], 0.799 (0.442)	p = .212, 1.57 [0.51, 4.57], 0.449 (0.575)
SN/VTA	p = .276, 1.54 [0.70, 5.08], 0.432 (0.510)	p = .016, 2.19 [1.10, 5.85], 0.785 (0.418)	p = .278, 1.52 [0.58, 6.51], 0.419 (0.594)
dlPFC	p = .054, 2.48 [1.11, 17.35], 0.909 (0.742)	p = .016, 2.28 [1.01, 6.18], 0.823 (0.511)	p = .271, 1.51 [0.57, 6.83], 0.415 (0.660)
Insula	p = .406, 1.40 [0.51, 4.08], 0.335 (0.513)	p = .028, 2.20 [1.07, 8.69], 0.789 (0.537)	p = .218, 1.58 [0.47, 5.31], 0.457 (0.692)

Table S24: Bootstrapped four-factor model: alcohol vs juice BOLD signal, EXT scores, CTQ scores, and sex predict follow-up high vs low alcohol use.

ROI	Alcohol vs Juice BOLD: p, OR [95% CI], B (SE)	EXT: p, OR [95% CI], B (SE)	CTQ: p, OR [95% CI], B (SE)	Sex: p, OR [95% CI], B (SE)
PCC	p = .002, 5.55 [2.28, 59.17], 1.714 (0.858)	p = .007, 2.74 [1.23, 32.52], 1.008 (52.309)	p = .413, 1.52 [0.06, 6.29], 0.419 (113.071)	p = .013, 0.12 [0.00, 0.64], -2.099 (219.004)
AS	p = .004, 6.62 [2.42, 51.02], 1.891 (100.830)	p = .007, 2.74 [1.23, 32.52], 1.008 (52.309)	p = .413, 1.52 [0.06, 6.29], 0.419 (113.071)	p = .013, 0.12 [0.00, 0.64], -2.099 (219.004)
vmPFC	p = .017, 1.227 [0.374, 6.507], 1.227 (32.828)	p = .017, 0.940 [0.073, 3.989], 0.940 (18.643)	p = .450, 0.449 [-3.826, 1.671], 0.449 (22.237)	p = .032, 0.20 [0.00, 0.79], -1.606 (15.641)
VS	p = .017, 5.90 [1.87, 90.99], 1.776 (101.429)	p = .023, 2.55 [1.05, 5.76], 0.935 (49.725)	p = .394, 1.68 [0.02, 6.64], 0.517 (22.610)	p = .013, 0.15 [0.00, 0.67], -1.899 (99.053)
ACC	p = .037, 2.64 [1.10, 15.78], 0.969 (0.620)	p = .014, 2.59 [1.15, 9.24], 0.951 (0.539)	p = .249, 1.56 [0.48, 6.53], 0.443 (0.628)	p = .005, 0.13 [0.003, 0.61], -2.014 (28.06)
SS	p = .115, 2.42 [0.88, 84.05], 0.882 (16.658)	p = .013, 2.52 [1.15, 34.09], 0.924 (9.438)	p = .486, 1.47 [0.08, 4.99], 0.385 (5.810)	p = .028, 0.19 [0.00, 0.85], -1.652 (9.844)
Amygdala	p = .058, 2.19 [0.84, 10.09], 0.782 (8.195)	p = .020, 2.37 [1.04, 11.78], 0.863 (31.990)	p = .374, 1.44 [0.19, 3.88], 0.368 (10.219)	p = .028, 0.21 [0.012, 0.89], -1.563 (44.860)
SN/VTA	p = .067, 2.19 [0.92, 15.17], 0.784 (1.821)	p = .021, 2.49 [1.06, 17.65], 0.914 (3.881)	p = .466, 1.33 [0.19, 4.66], 0.286 (2.472)	p = .012, 0.17 [0.003, 0.66], -1.778 (6.883)
dlPFC	p = .054, 2.48 [1.11, 17.35], 0.909 (0.742)	p = .016, 2.28 [1.01, 6.18], 0.823 (0.511)	p = .271, 1.51 [0.57, 6.83], 0.415 (0.660)	p = .078, 0.282 [0.020, 1.165], -1.264 (76.757)
Insula	p = .406, 1.40 [0.51, 4.08], 0.335 (0.513)	p = .028, 2.20 [1.07, 8.69], 0.789 (0.537)	p = .218, 1.58 [0.47, 5.31], 0.457 (0.692)	p = .062, 0.25 [0.009, 0.97], -1.372 (2.444)

Additional Methodological Details

Given the modest sample size ($n = 44$), regression coefficients, standard errors, p-values, and 95% confidence intervals were also estimated using non-parametric bootstrapping with 1,000 resamples (percentile method, simple sampling) to improve the robustness of inference and reduce reliance on parametric assumptions. This approach resamples the dataset with replacement to generate an empirical distribution of parameter estimates, allowing more accurate estimation of standard errors and CIs in small samples (1).

The Box-Tidwell transformation was applied to assess the assumption of linearity between the continuous predictors (BOLD response, CTQ, EXT scores) and the logit of the binary outcome (high vs low alcohol use) (2). Interaction terms (e.g., BOLD x ln (BOLD)) were included in the logistic regression model. A Bonferroni correction was applied to account for multiple comparisons across all seven terms (BOLD, EXT, CTQ, BOLD*ln_BOLD, EXT*ln_EXT, CTQ*ln_CTQ, and the constant). The interaction terms were non-significant for all tests ($p \geq 0.232$), indicating that the continuous predictors were linearly related to the logit.

Receiver operating characteristic (ROC) curves were generated for each model to evaluate performance. Sensitivity (true positive rate) was plotted on the y-axis, and 1-specificity (false positive rate) was plotted on the x-axis. The area under the curve (AUC) quantified the model's overall discriminatory ability, with an AUC approaching 1 indicating strong discrimination and an AUC near 0.5 indicating poor performance (3).

Likelihood ratio tests (LRTs) were used to evaluate whether the inclusion of CTQ scores, ROI-specific BOLD responses (Alcohol vs. Juice or Alcohol vs. Water), and sex improved model fit compared to models with fewer predictors. Comparisons spanned one- to four-factor models depending on the outcome (high alcohol use or DSM-5 diagnoses) and the specific

combination of predictors (e.g., three- (EXT scores + CTQ scores + fMRI BOLD) vs. two-factor models (EXT scores + fMRI BOLD)) (3). The LRT compares model fit by testing the difference in -2 log-likelihood values (−2LL) between nested models:

$LR = -2LL_{reduced} - (-2LL_{three\text{-}full})$

The resulting test statistic follows a chi-square (χ^2) distribution, with degrees of freedom equal to the difference in the number of estimated parameters. A significant result ($p < 0.05$) indicates that the more complex model provides a significantly better fit.

For continuous follow-up AUDIT scores, nested multiple linear regression models were compared using the change in explained variance (ΔR^2) and the associated F-change statistic. Specifically, for each ROI we compared a reduced two-factor model (CTQ + EXT) to a full three-factor model (CTQ + EXT + ROI-specific alcohol cue-evoked BOLD response). In follow-up analyses, sex was added as an additional predictor (four-factor model) and model improvement was evaluated using ΔR^2 and F-change relative to the corresponding three-factor model. A significant F-change ($p < .05$) indicated that the added predictor explained incremental variance in follow-up AUDIT scores beyond the reduced model (4).

Regions of Interest

Functional subdivisions of the striatum were defined as previously described (5). The midbrain region (SN and VTA) corresponded to the mask provided by Dr. Adcock's Laboratory at Duke University (6). The ACC and dlPFC were derived from the third version of the Automated Anatomical Labelling atlas (AAL3) (7). The dlPFC ROI was created by combining the left and right superior frontal gyrus and middle frontal gyrus. For the vmPFC, we created two 6-mm spheres, one per hemisphere, that were later combined, around peak coordinates (x = 4, y

= 46, z = -9) from a meta-analysis in drug users that identified regions consistently activated by drug-related cues (8).

References

1. Efron B, Tibshirani RJ (1994): *An Introduction to the Bootstrap*. New York: Chapman and Hall/CRC. https://doi.org/10.1201/9780429246593
2. Box GEP, Tidwell PW (1962): Transformation of the Independent Variables. *Technometrics* 4: 531–550.
3. Hosmer DW, Lemeshow S, Sturdivant RX (2013): *Applied Logistic Regression*, 1st ed. Wiley. https://doi.org/10.1002/9781118548387
4. Cohen J, Cohen P, West SG, Aiken LS (2003): *Applied Multiple Regression/Correlation Analysis for the Behavioral Sciences, 3rd Ed*. Mahwah, NJ, US: Lawrence Erlbaum Associates Publishers, pp xxviii, 703.
5. Martinez D, Slifstein M, Broft A, Mawlawi O, Hwang D-R, Huang Y, *et al.* (2003): Imaging human mesolimbic dopamine transmission with positron emission tomography. Part II: amphetamine-induced dopamine release in the functional subdivisions of the striatum. *J Cereb Blood Flow Metab* 23: 285–300.
6. Murty VP, Shermohammed M, Smith DV, Carter RM, Huettel SA, Adcock RA (2014): Resting state networks distinguish human ventral tegmental area from substantia nigra. *Neuroimage* 100: 580–589.
7. Rolls ET, Huang C-C, Lin C-P, Feng J, Joliot M (2020): Automated anatomical labelling atlas 3. *Neuroimage* 206: 116189.

8. Chase HW, Eickhoff SB, Laird AR, Hogarth L (2011): The neural basis of drug stimulus processing and craving: an activation likelihood estimation meta-analysis. *Biol Psychiatry* 70: 785–793.
